# Supplementary material for: Perceptions and experiences of daily and long-acting pre-exposure prophylaxis (PrEP) among MSM in India
Source: PLOS Glob Public Health. 2024 Jan 17;4(1):e0001477. doi: 10.1371/journal.pgph.0001477 (PMC10793913; doi:10.1371/journal.pgph.0001477)
Supplement: Attachment — Submitted filename: Response to reviewers.docx [file pgph.0001477.s001.docx]

**In the ethics statement in the Methods, you have specified that verbal consent was obtained. Please provide additional details regarding how this consent was documented and witnessed, and state whether this was approved by the IRB**

We discuss the informed consent process in the participant recruitment section. We have now provided additional details and made necessary edits on page 4 Line 14 to 18. The verbal consent process happened between the participant and the interviewer, hence there were no witnesses. Also, since the interview was recorded only after the participant consented to participate and to record, it was not documented. This process was approved by IRB. The revised text is copied below for your reference.

*All interviews were conducted through Health Insurance Portability and Accountability Act (HIPPA) compliant teleconferencing (Zoom). Before the start of each interview, the interviewer read and discussed the IRB approved informed consent form section by section and answered the questions participants had. Participants were also given more time if required to make the decision. Once the participant decided to take part in the study, they provided a verbal consent to participate in the study and to audio record the interview.. The interview was started after the consent was given to participate in the study and to record the interview.*

**Please send a completed ‘Competing Interests’ statement, including any COIs declared by your co-authors. If you have no competing interests to declare, please state “The authors have declared that no competing interests exist”. Otherwise please declare all competing interests beginning with twhe statement “I have read the journal’s policy and the authors of this manuscript have the following competing interests:”**

We have provided the complete competing interests’ statement in this revised manuscript. Please let us know if anything else is needed here.

**Suggest reviewing for formatting and copyediting, including spacing, punctuation and spelling. There are some inconsistencies and typos throughout.**

Thank you for this suggestion. We have edited the manuscript throughout to improve for these areas.

**Introduction**

**Suggest adding the year for the HIV sentinel surveillance data in the text body. (“The HIV sentinel surveillance data reported a decrease in HIV prevalence in general population and among MSM at .2% and reported and 3.3% respectively.”) Otherwise it is unclear whether there was a decrease compared to NACO’s data or if they are just from different sources.**

We have added the year and also updated NACO reported prevalence with more recent data. We have also clarified that the survey is a different source. Since the new NACO source doesn’t have MSM specific prevalence, we have made other edits and added more citations to establish the context on page 3 from line 1 to 15. The revised text is copied below for your reference.

*National AIDS Control Organization (NACO) reported the HIV prevalence in 2021 as 0.21% in 2021 among all adult population (Age 15-49) in India*[*(1)*](https://sciwheel.com/work/citation?ids=15285507&pre=&suf=&sa=0&dbf=0)*. Separately, the 2021 HIV sentinel surveillance data reported similar HIV prevalence of .2%*[*(2)*](https://sciwheel.com/work/citation?ids=15285523&pre=&suf=&sa=0&dbf=0)*. At the same time, the sentinel survey also reported HIV prevalence among men having sex with men (MSM) as 3.3%*[*(2)*](https://sciwheel.com/work/citation?ids=15285523&pre=&suf=&sa=0&dbf=0)*. Several other studies in literature also observe that MSM group have higher prevalence and burden of HIV in India*[*(3–5)*](https://sciwheel.com/work/citation?ids=15287658,916481,4198346&pre=&pre=&pre=&suf=&suf=&suf=&sa=0,0,0&dbf=0&dbf=0&dbf=0)*.*

**Endnote #4: consider a more recent source, if available.**

Thank you for this suggestion. We have replaced the previous source with a more recent one and also edited the text on page 3 line 20 to 24. The revised text is copied below:

*Pre-exposure prophylaxis (PrEP) is an effective HIV prevention method in addition to condom use among populations at higher risk of HIV acquisition like MSM or men in same-gender relationships*[*(6)*](https://sciwheel.com/work/citation?ids=13547998&pre=&suf=&sa=0&dbf=0)*. A systematic review that included 15 randomized control trials found a risk ratio of 0.25 for MSM population and also found that PrEP efficacy was significantly associated with adherence*[*(7)*](https://sciwheel.com/work/citation?ids=15096258&pre=&suf=&sa=0&dbf=0)*.*

**Methods**

**The rest of this sentence seems to be cut off; consider revising: “…our interview guide first explored the participants knowledge of PrEP and initial impressions of PrEP followed by an exploration of their key motivators to initiate PrEP and (including what aspects of PrEP were appealing to them).”**

Thank you for highlighting this. We have corrected this. The sentence now finishes with ‘motivators to initiate PrEP’ and is complete.

**Suggest condensing the paragraph beginning “Post that…” to improve readability and comprehension. It does not need to present all of the questions asked, but rather provide a sense of the major themes covered in the interview guide.**

Thank you for this suggestion. We have now listed overall themes and removed some parts of the paragraph to condense it. These changes are made from page 4 line 37. The revised text is copied below for your reference.

*We nested questions about barriers and concerns within these themes to learn what kind of barriers or concerned participants faced and how they were able to overcome those. Other themes included perceptions around costs, sexual behavior while on PrEP, adherence and long-term goals with PrEP and LA PrEP*

**• Suggest removing or clarifying the question mark in the middle of this sentence: “All interview recordings in English were transcribed verbatim? by professional transcribers.”**

The additional question mark was an error. We have removed it now.

**Results**

**As you’ll see in the comments below, there are areas of the Results that provide potential hypotheses but are not fully supported by the primary data and would be better in the Discussion section with corresponding citations.**

We agree with the reviewers’ comments and have made suitable changes to address this in relevant sections. We have provided more details next to the specific feedback provided further below.

**The first couple of sub-sections with qualitative findings could be condensed and tell more of a story to improve flow and comprehension. The latter sections do this well. See specific suggestions below.**

Thank you for this suggestion. We have condensed it to keep the focus on the most important findings and re-arranged text to improve flow and comprehension.

**Key Motivators to Use PrEP**

**In this section, it is unclear which motivators came out most strongly and which were only cited by one or few participants. Suggest clarifying which were raised by more participants so that readers have a sense of the greatest motivators, and perhaps narrow the focus of this section.**

We thank the reviewers for this suggestion. We have used ‘few’, ‘some’ and ‘many’ to indicate if a theme was elicited by less or more participants. We have removed some parts on page 7 to narrow down the focus.

**Graduate/post-graduate information was included in the sentence prior to: “Participants mostly resided in urban metropolitan areas of India, were either graduate or post graduate, belonged to general caste, and had an income.” Consider removing here.**

Thank you for this suggestion. We have now removed it.

**Suggest moving the following to the “Participant Demographics” section: “13 participants were using PrEP at the time of the interview, 4 participants had used PrEP in the past and were no longer using it, and 1 participant reported using PrEP on Demand model. The median duration on PrEP was 10.5 months (minimum being 1 month and maximum being 48 months).”**

Thank you for this suggestion. We have moved this sentence to the participants demographics now.

**Is this sentence speculative or did it come up in the primary research? “Such fear could be possible because of lack of proper knowledge about HIV and the lifesaving antiretroviral treatments that make living with HIV manageable and provide similar quality of life to those who are HIV negative.” Suggest keeping this section to only findings supported by the data collected. If there is supporting evidence from other published literature, this sentence could be moved to the Discussion section.**

­­­­

Thank you for this suggestion. Considering the overall review of narrowing the focus and condensing this section, we have decided to remove this speculative thought and the quote.

**The following sentence would read better in the Discussion section with supporting evidence, as it appears to extrapolate based on the quote but is not what the participant stated. “Participant sharing this provides an insight into the existing discrimination that people living with HIV have to face when it comes to seeking sexual or romantic partners, something that could further lead to reduced self-confidence, isolation and adverse mental health outcomes among those who live with HIV.”**

Thank you for this suggestion. Considering the overall review of narrowing the focus and condensing this section, we have decided to remove this speculative thought.

**Add a citation to the following sentence and consider moving it to the Discussion section: “This touches upon the lives of many bisexuals and closeted gay individuals who maintain sexual relations with other male partners but prefer to marry a woman, have kids and start a family to meet the social norms and expectations.”**

Thank you for this suggestion. We have moved this to the discussion section. As compared to other speculative thoughts that we removed, we feel this is important to Indian context and hence we have decided to keep it. We have also added relevant citations to support this.

**Experiences Accessing PrEP**

**Suggest further grouping some of these findings to improve flow and comprehension, focusing on the findings that came out most strongly.**

Thank you for this suggestion. We have re-arranged the text substantially and added subheadings so that it is easy to follow. We also deleted some parts which we felt were less relevant or were repetitive to keep the focus on findings that came out strongly.

**Add supporting evidence to this sentence if available, or expand to indicate that this has been seen in other settings: “…since PrEP could be equated with promiscuous behavior.” Consider moving to the Discussion section.**

We have decided to remove it since the text preceding this reads complete in conjunction with the quote right above.

**Suggest combining the following sentence with the one prior to it (above the quote, beginning with “Home delivery...”) as they are saying similar things: “Participants appeared to enjoy and appreciate the convenience and option to either get it home delivered or to pick up from the pharmacy.”**

Thank you for this suggestion. We have combined these two sentences on page 11 line 36 to 38.

**Consider this as an opening paragraph to orient the reader: “Participants’ experiences differed as per the route through which they accessed PrEP. However, the most common route to seek PrEP was through NGOs that offered PrEP at subsidized or no cost.”**

Thank you for this suggestion. We have used this as the opening paragraph for this section on page 8 line 45.

**Concerns and Dislikes around PrEP**

**Consider rephrasing “indulge in risky sexual behaviors” throughout this paragraph as this could imply a value judgement and the field is moving away from risk-based framing.**

Thank you for this important suggestion. We have made the required changes on page 14 line 4 to 8. The revised text is copied below

*The above indicates that participant’s propensity to not use condoms increases when under the influence of alcohol or other substances, and if they are on PrEP. It is worth noting that the participant did not consider using PrEP alone as a safe sex practice. Overall, in our study, participants reported minor concerns and dislikes – none of which deterred them from continuing to use PrEP. The increased propensity to not use condoms also didn’t results in discontinuation of PrEP.*

**PrEP Use and dual protection**

**Is there a citation/supporting literature you can add here?: “This alludes to the increased use of aphrodisiacs in urban Indian MSM communities…”**

This was a finding from some of the other quantitative research conducted by some of the co-authors. That research, however, is unpublished for now and is in the review process. We have removed this sentence.

**Acceptability of Long-Acting PrEP**
**Suggest changing “muscular injection” to “intramuscular injection.”**

Thank you for this suggestion. We have made appropriate correction as recommended.

**Discussion**

**Suggest including the number of participants in some of the studies referenced, particularly those with larger numbers of participants, to indicate how applicable or prevalent certain findings are.**

Thank you for this suggestion. We have added sample size for the cited studies. Many of them, however, are qualitative and hence we have not included sample size for those.

**Add citations to the following sentences if available: “The supreme court of India decriminalized same-sex relationships in 2018, but the battle for equal rights to marry continues in India. These reflections also contradict the usual misconceptions and stereotypes about gay community being promiscuous.”**

We have added several relevant citations for the given sentences.

**Last paragraph on on-demand PrEP: consider adding that your study found that discontinuation of PrEP was linked to periods of sexual activity as another reason to raise awareness for event-driven PrEP use.**

Thank you for this suggestion. We have added this in the discussion on page 23 line 13 and 14

*Along with that, considering our research found that discontinuation of PrEP was linked to periods of no sexual activity, more awareness about event driven PrEP could prove to be further beneficial.*

**Conclusion**

**Suggest unpacking and clarifying what this question is implying: “However, at the same time, would the community at large continue to show high adherence if PrEP becomes cheaper and further available?” Is it saying that making PrEP more accessible would reduce adherence, and if so, why is that the takeaway based on what was presented in this study?**

Thank you for this suggestion. We have removed this and taken a different approach to our conclusion.

*Our study documents real-world experiences of MSM using PrEP in India and provides critical insights that can inform further scale up of PrEP in India in the MSM population. We learn that MSMs are early adopters of oral PrEP in India and are reporting high adherence. Key considerations for further scale up for PrEP in India would include more awareness about PrEP in the community and further reducing out-of-pocket expenses to access PrEP. Further, capacities of NGOs providing services to the community should be strengthened and such NGOs should be leveraged for providing PrEP to the community. The interest in LA PrEP should be harnessed and efforts to get LA PrEP in India should be accelerated.*
