# Supplementary material for: Perceptions and experiences of daily and long-acting pre-exposure prophylaxis (PrEP) among MSM in India
Source: PLOS Glob Public Health. 2024 Jan 17;4(1):e0001477. doi: 10.1371/journal.pgph.0001477 (PMC10793913; doi:10.1371/journal.pgph.0001477)
Supplement: Attachment — Submitted filename: Response to reviewers_261123.docx [file pgph.0001477.s002.docx]

**While noting that the revision included a thorough copyedit, I suggest doing an additional review for consistency, as there are still some typos, missing periods, and other formatting errors throughout.**

We have made a number of formatting, spelling, spacing and typo related changes in the final cleaned file and not in tracked version.

**Methods**
**• Please correct the spelling of “HIPPA” to “HIPAA.”**

Thank you, we have corrected the spelling.
**Results**

**Participant Demographics**
**• In Table 1, correct misalignment of participant numbers in the righthand column. In addition, consider changing “Top (Prefer to penetrate)” and “Bottom (Prefer to get penetrated)” to “Top (Prefer insertive anal sex)” and “Bottom (Prefer receptive anal sex),” respectively.**

We have changed the text in bracket for both Top and Bottom. We have also aligned the numbers but we note that they move when converting to other formats. We are hoping that journal would take care of it when typesetting it.

**Key Motivators to Use PrEP**
**• Consider revising “these real conversations” to “these conversations” or “conversations covering these issues.”**

We have removed ‘real’ from the text. Now it reads ‘these conversations’ as suggested.

**Experiences Accessing PrEP**
**• For “Sources of information,” suggest starting with lines 20-42 (the paragraphs beginning: “Participants identified multiple sources from which they first heard about oral PrEP...”). This would reorganize the section in more sequential order, starting with where participants learned about PrEP followed by their experiences once they tried to access it. To this end, I suggest moving the sentence, “Few participants felt that it was hard to find PrEP in India when they started searching and that PrEP was expensive, which appeared to be one of the barriers to initiating or to stay on PrEP,” and corresponding quote to the end of the Sources of Information section.**

We have moved both sentences as suggested by the reviewer.

**• To improve flow, consider moving the sentence “Few participants reported providers to be rude or robotic” (line 36 on p. 10) to appear alongside the corresponding data on p. 11, beginning with the sentence, “One participant felt that their provider was robotic and that the provider was in rush…”**

We have removed it from the line 36 and page 10. However, the text below the quote already mentions ‘robotic’, hence we are not copying it back to avoid duplication.

**• Suggest combining the following two sentences into one, as they are making similar statements: “Home delivery seemed to be the preferred mode for accessing PrEP, along with pick up from pharmacies. Participants appeared to enjoy and appreciate the convenience and option to either get it home- delivered or to pick- up from the pharmacy.”**

We have merged this sentence as following:

Participants appeared to enjoy the convenience of home delivery, making it a preferred option; and also appreciated the option to pick- up from the pharmacy.

**Concerns and Dislikes around PrEP**
**• Suggest changing “initial” to “initiated” in the following sentence: “However, very few reported any side effects once they initial oral PrEP.”**

We have corrected this.

**Adherence and Trust with PrEP**
**• In lines 9-41 on p. 13, trust is described as being fostered before a participant initiates PrEP. Consider moving this section to “Experiences Accessing PrEP” where sources of information and experiences with providers are discussed.**

We have removed the part of text corresponding to trust to ‘Experiences accessing PrEP; after sources of information to make it more sequential. However, we have kept Adherence section at its original position.

**Cost and Affordability**
**• The following quote is missing attribution to a participant: “So I wouldn't say it's like super cheap also but it's affordable for me and but the concern could be that, for some people out there it could be too expensive. Even at the subsidized rate.”**

We have added the attribution.

**Community and Peers’ Reactions to PrEP Use**
**• Lines 22-46 on p. 16 pertain less to the wider community and are more focused on condom negotiation with sexual partners. They could therefore be moved to the “PrEP Use and Dual Protection” section or cut if duplicative.**

Sexual partners are also the peers, and these lines correspond to that. Hence, we prefer to keep these lines in the current section. We don’t feel that the PrEP use and dual protection is the right position for it.

**• The following sentence is speculative; suggest removing or moving to the Discussion section: “This could be because of a lack of access on their part but at the same time having the desire to indulge in unprotected sex.”**

We have removed this sentence.
